# Involved-Field Radiation Therapy for Patients With Unresectable Pancreatic Adenocarcinomas: Failure Pattern Analysis

**DOI:** 10.7759/cureus.48106

**Published:** 2023-11-01

**Authors:** Ricardo C Fogaroli, Douglas G Castro, Maria L Silva, Antonio Cassio A Pellizzon, Guilherme R Gondim, Michael J Chen, Henderson Ramos, Elson S Neto, Carolina H Abrahão

**Affiliations:** 1 Radiotherapy, A.C. Camargo Cancer Center, São Paulo, BRA; 2 Radiation Oncology, A.C. Camargo Cancer Center, São Paulo, BRA

**Keywords:** distant metastases, failure patterns, involved-field irradiation, palliative radiation therapy, pancreatic-biliary cancer

## Abstract

Introduction

Unresectable pancreatic tumors are frequently diagnosed. Initial treatment is carried out with chemotherapy. Eventually, in selected cases, radiotherapy may be used to improve local control rates and relieve the symptoms. The volume of radiotherapy treatment fields is the subject of controversy in the literature. The use of involved fields with the gross tumor volume encompassing the primary tumor and lymph nodes considered clinically positive is associated with a lower rate of side effects, but can lead to a higher rate of regional loco failures, especially in regional lymph nodes. The purpose of this article is to analyze the failure pattern of chemotherapy and involved-field radiation therapy (IFRT) for treating patients with unresectable pancreatic adenocarcinomas.

Methods

Clinical records of thirty consecutive patients treated from March 2016 to June 2020 for unresectable pancreatic adenocarcinoma were analyzed. The patients were treated with initial systemic chemotherapy (median: 6 cycles) with regimens based on gemcitabine or oxaliplatin-irinotecan (folfirinox/folfox) followed by radiotherapy (total dose of 50-54 Gy/with fractionation of 2 Gy/day). The patients were treated with IFRT. Local failure (LF) was defined as an increase in radiographic abnormality within the planning target volume (PTV). Elective nodal failure (ENF) was defined as recurrence in any lymph node region outside the PTV. Any other failure was defined as distant failure (DF).

Results

The median age of the patients was 68 years (range: 44-80 years); 20 patients (66.7%) were men, and 11 (36.6%) and 19 (63.4%) patients presented with tumors of stage II and III, respectively. Most patients (63.3%) had tumors in the pancreatic head. The median survival was 17.2 months. Tumor recurrences were classified as LF, DF, LF and DF in 7 (23.3%), 17 (56.7%), and 5 (16.7%) patients, respectively. Only one patient (3.3%) had both LF and ENF. No severe side effects related to radiotherapy were reported.

Conclusion

The use of IFRT did not cause a significant amount of ENF, besides presenting low morbidity, which is of special importance for patients with locally advanced tumors or low performance status. The predominant failure pattern was distant metastases.

## Introduction

Despite the progress achieved in various treatment modalities, pancreatic cancer remains one of the leading causes of cancer-related mortality. During 2022, approximately 62,210 diagnoses of this neoplasm were made in the United States, with an estimated 49,380 deaths. Pancreatic cancer is the fourth most common cause of cancer-related death among American men, after lung, prostate, and colorectal cancers [1].

At diagnosis, 20% of patients have resectable tumors, 50% are diagnosed with metastatic disease, and 30% with locally advanced tumors considered unresectable [2]. Patients with unresectable tumors are generally elderly and present with debilitating clinical conditions. Hence, the treatment should be judicious and individualized, aiming at maximum clinical benefit with minimal associated morbidity.

The preferred initial treatment for this specific group of patients is chemotherapy whenever possible. Radiotherapy after chemotherapy may be considered for some carefully selected patients with favorable clinical conditions who have not developed metastases. Radiotherapy can improve local control and reduce morbidity caused by local symptoms of tumor progression, thereby improving the quality of life [3]. However, an important aspect to be considered when choosing to include radiotherapy is clinical complications that may occur owing to the proximity of high-risk organs, especially the stomach and small intestine. A critical factor to consider is the treatment volumes, with the inclusion of elective lymphatic drainage or with involved-field radiation therapy (IFRT), with volumes restricted to the primary lesion and compromised lymph nodes. This subject is controversial in the literature. This study analyzed the failure patterns in 30 patients with unresectable tumors treated using chemotherapy followed by IFRT to evaluate their effectiveness.

## Materials and methods

This study retrospectively analyzed the clinical records of 30 patients diagnosed with pancreatic adenocarcinoma, with tumors considered unresectable, and treated from March 2016 to June 2020. All cases were discussed in a tumor board comprising surgeons, clinical oncologists, radio-oncologists, radiologists, anesthesiologists, and palliative-care specialists before making a therapeutic decision. The patients were evaluated with computed tomography (CT) scans of the chest, pelvis, and abdomen under a protocol for the pancreas; magnetic resonance imaging of the upper abdomen if CT was inconclusive; CA 19‑9 after adequate drainage of the biliary tract; and complementary tests, such as blood count, aspartate aminotransferase, alanine aminotransferase, bilirubin (total and fractions), gamma-glutamyl transferase, alkaline phosphatase, urea, and creatinine. The tumor stage, nodal stage, and clinical prognostic groups were classified according to the International Union against Cancer (UICC) tumor-node-metastasis (TNM) classification, eighth edition, 2017.

All patients received initial treatment with initial systemic chemotherapy. The chemotherapy regimens used were based on gemcitabine (1000 mg/m^2^, intravenous (IV), weekly), gemcitabine-capecitabine (capecitabine 830 mg/m^2^ twice a day, days 1-21; gemcitabine 1000 mg/m^2^, intravenous (IV) days 1, 8, 15), folfirinox (oxaliplatin 85 mg/m^2^, irinotecan 180 mg/m^2^, 5-fluorouracil 400 mg/m^2^ (IV), 2400 mg/m^2^ via infusor device, over 46 hours, every 14 days for a maximum of 12 cycles), folfox (oxaliplatin 85 mg/m^2^ IV, 5FU bolus 400 mg/m^2^, then by 5FU continuous infusion 2400 mg/m^2^ in 46 hours). The number of cycles was determined after obtaining a maximum benefit, limiting toxicity for treatment continuation, or both. At the end of chemotherapy, the patients were restaged with computed tomography (CT) scans of the chest, pelvis, and abdomen under a protocol for the pancreas; CA 19‑9 and complementary tests, such as blood count, aspartate aminotransferase, alanine aminotransferase, bilirubin (total and fractions), gamma-glutamyl transferase, alkaline phosphatase, urea, and creatinine, and when they did not present clinical evidence of distant metastasis and were in a satisfactory clinical condition, they were referred for radiotherapy after a new discussion in the tumor board. All patients were treated with IFRT. The gross tumor volume (GTV) included the primary residual tumor after chemotherapy and the lymph node chains considered positive on imaging at initial staging. For planning target volume (PTV), margins of 2 cm in the craniocaudal axis and 1.0-1.5 cm in the other axes were used. The dose used ranged from 50 to 54 Gy with daily fractionation of 2 Gy according to the dose tolerance protocol for normal organs adjacent to the PTV. The dose restrictions for normal organs were: for the kidney no more than 30% of the total volume can receive a dose equal to or greater than 30 Gy, for the stomach, duodenum and jejunum the maximum dose was 55 Gy, for the liver the mean dose cannot exceed 30 Gy and for the spinal cord the maximum dose to a volume of at least 0.03 cc must be less than or equal to 45 Gy. All patients underwent a clinical review consultation and nutritional status assessment during the treatment.

The patients were followed up with abdominal CT and tumor markers every three months in the first and second years and every six months from the third year of follow-up and with PET-CT when indicated. All patients were followed up until the date of death, and the date of the beginning of the follow-up was considered the first day of chemotherapy. Local failure (LF) was defined as an increase in radiographic abnormality within the PTV. Elective nodal failure (ENF) was defined as recurrence in any lymph node region outside the PTV. Any other failure was defined as distant failure (DF).

Study endpoints and statistical analysis

The study endpoints of LF, ENF, and DF were evaluated. Initially, a descriptive analysis of the variables was performed, in which the absolute (n) and relative (%) frequency distributions were presented for qualitative variables, and the main summary measures, such as mean, standard deviation, median, and minimum and maximum values, were calculated for quantitative variables. To assess associations between categorical variables, the chi-square test or Fisher’s exact test was used when appropriate. The Kaplan-Meier estimator was considered when determining the survival curve. A 5% significance level was adopted, and statistical analyses were performed using SPSS version 28 (IBM Corp., Armonk, NY, USA).

## Results

A predominance of male patients with clinical stage III pancreatic head tumors was observed. The other clinical characteristics of the patients are listed in Table 1.

**Table 1:** Patient characteristics TNM: The tumor stage, nodal stage, and clinical prognostic groups were classified according to the International Union against Cancer (UICC) tumor-node-metastasis (TNM) classification, eighth edition, 2017

Variables	Patients (n = 30)	%
Sex		
Male	16	53.3
Female	14	46.7
Age (years)		
Median	69.5 (range: 51-80 years)	
TNM stage		
T3N0M0	8	26.6
T3N1M0	3	10
T4N0M0	14	46.6
T4N1M0	5	16.8
Clinical stage		
II	11	36.6
III	19	63.4
Tumor site		
Head	16	53.3
Body	4	13.4
Head/body	3	10
Body/tail	2	6.7
Uncinate process	2	6.7
Body/uncinate process	1	3.3
Tail	1	3.3
Body/tail	1	3.3

The characteristics of the chemotherapy and radiotherapy treatments are presented in Table 2.

**Table 2:** Treatment characteristics Gy: Gray;  folfirinox: leucovorin, calcium (folinic acid), fluorouracil, irinotecan hydrochloride, and oxaliplatin; folfox: leucovorin, calcium (folinic acid), fluorouracil, and oxaliplatin; 5FU: Fluorouracil; RXT: radiotherapy; IMRT: intensity-modulated radiotherapy.

Variables	Patients (n = 30)	%
Radiotherapy dose (Gy)		
50	13	43.3
54	17	56.7
Radiotherapy technique		
3D	18	60
IMRT	12	40
Chemotherapy		
Median: 6 cycles (range: 4-20 cycles)		
Gemcitabine/capecitabine	2	6.7
Gemcitabine	8	26.6
Folfirinox	9	30
Folfirinox/folfox	4	13.4
Folfox	7	23.3
Chemotherapy with RXT		
Capecitabine	15	50
5FU	9	30
None	6	20

**Figure 1:**
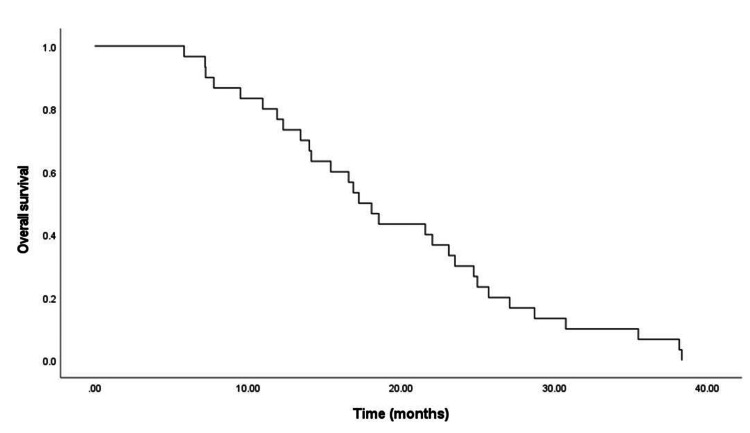
Overall survival for the entire cohort

All patients died because of tumor progression and were followed up until the date of death. Overall survival ranged from 7.2 to 38.1 months, and the median survival was 17.2 months. The overall survival curve is shown in Figure 1.

Failure pattern

Regarding the main site of disease progression, DF was observed in isolation in 17 patients and in combination with LF in five patients. The sites of disease progression for all patients are shown in Table 3. ENF was observed in only one patient in combination with LF. The sites affected in cases of DF are shown in Table 4.

**Table 3:** Treatment failure patterns

Failure site	Patients (n = 30)	%
Distant failure	17	56.7
Local failure	7	23.3
Local failure and distant failure	5	16.7
Local failure and elective nodal failure	1	3.3

**Table 4:** Sites affected in cases with distant failure

Metastasis site	Patients (n)
Liver	11
Lung	11
Peritoneum	9
Subcutaneous	1
Adrenal	1

No statistically significant correlation in univariate analysis was observed for the variables tumor, lymph node status, clinical stage, radiotherapy dose, and number of chemotherapy cycles in relation to LF or DF (Tables 5, 6).

**Table 5:** Associations of treatment-related variables with local failure (univariate analysis) Gy: Grays; Cht: chemotherapy

Treatment-related variables	With local failure (n, %)	Without local failure (n, %)	p
Tumor			0.45
T4	07 (53.8%)	12 (70.5%)	
T3	06 (46.2%)	05 (29.5%)	
Total	13 (100%)	17 (100%)	
Lymph nodes status			0.69
N1	04 (30.8%)	04 (23.5%)	
N0	09 (69.2%)	13 (76.5%)	
Total	13 (100%)	17 (100%)	
Clinical stage			0.45
II	06 (46.2%)	05 (29.5%)	
III	07 (53.8%)	12 (70.5%)	
Total	13 (100%)	17 (100%)	
Radiotherapy dose (Gy)			0.92
50	05 (38.5%)	08 (47.1%)	
54	08 (61.5%)	09 (52.9%)	
Total	13 (100%)	17 (100%)	
Cht cycles			0.67
≤ 6	08 (61.5%)	08 (47.1%)	
> 6	05 (38.5%)	09 (52.9%)	
Total	13 (100%)	17 (100%)	

**Table 6:** Associations of treatment-related variables with distant failure (univariate analysis) Gy: Gray; Cht: chemotherapy

Treatment-related variables	With distant failure (n, %)	Without distant failure (n, %)	p
Tumor			1.00
T4	14 (63.6%)	05 (62.5%)	
T3	08 (36.4%)	03 (37.5%)	
Total	22 (100%)	08 (100%)	
Lymph nodes status			0.64
N1	05 (22.7%)	03 (37.5%)	
N0	17 (77.3%)	05 (62.5%)	
Total	22 (100%)	08 (100%)	
Clinical stage			1.00
II	08 (36.4%)	03 (37.5%)	
III	14 (63.6%)	05 (62.5%)	
Total	22 (100%)	08 (100%)	
Radiotherapy dose (Gy)			0.69
50	09 (40.9%)	04 (50%)	
54	13 (59.1%)	04 (52%)	
Total	22 (100%)	08 (100%)	
Cht cycles			1.00
≤ 6	12 (54.5%)	04 (50%)	
> 6	10 (45.5%)	04 (50%)	
Total	22 (100%)	08 (100%)	

Morbidity

Twenty-two patients presented with toxicity symptoms during radiotherapy treatment. There were six cases of diarrhea, six cases of myelotoxicity, six cases of nausea, three cases of vomiting, and one case of cholangitis. All cases were resolved with clinical treatments, and no deaths were caused by these complications in any of the patients.

## Discussion

Some aspects related to the use of radiotherapy for treating unresectable pancreatic lesions are controversial.

Indication

The use of chemoradiation after induction chemotherapy for patients is a therapeutic option to be considered in specific clinical situations, and the aim is to reduce the risk of local tumor progression and relieve the symptoms. This approach is generally recommended for patients in whom it is highly unlikely that the lesion will become resectable owing to arterial impairment or for those definitively not considered as candidates for surgery after completion of induction chemotherapy. An analysis of patients with this clinical condition by the US National Cancer Data Base showed favorable results when the patients were treated using radiotherapy with conventional fractionations or using stereotactic body radiation therapy (SBRT) [4].

The influence of adding radiotherapy to treatments that exclusively involve chemotherapy on patient survival is doubtful. The SCALOP phase‑II trial analyzed the outcomes of patients treated with gemcitabine-based chemotherapy or chemoradiation with capecitabine-based chemotherapy. There was no statistically significant difference in overall survival and disease-free survival between the two groups. Similar to the median survival results obtained in our study, with chemotherapy treatments followed by radiotherapy, the median survival was 17.6 months in the group receiving chemoradiation and 12 months in the group treated with chemotherapy alone [5]. In the phase‑III LAP‑07 study involving 269 patients treated using chemoradiation with gemcitabine or chemotherapy alone with gemcitabine and erlotinib, there was no difference in survival between the two treatments (hazards ratio 1.03; 95% confidence interval [CI] 0.79-1.34, p = 0.83). However, the local progression rate was favorable in the case of treatment using chemoradiation when compared with the group treated using chemotherapy alone, with 34% and 65% rates, respectively (p < 0.0001) [6].

Vornhülz et al. conducted a systematic review of the use of SBRT for treating locally advanced tumors of the pancreas, with an emphasis on symptom improvement. This review analyzed 11 studies with 292 patients treated using 3-6 fractions with a dose of 4-15 Gy per fraction, and 73% of the patients underwent chemotherapy prior to radiotherapy. The authors observed significant improvement in pain, weight loss, and nausea [7].

Volume

The volume to be treated in patients with pancreatic tumors that are not candidates for surgery is controversial in the literature, especially regarding the inclusion of elective lymphatic drainage. In favor of this inclusion, there is a high incidence of lymph node involvement in locally advanced tumors. The potential ability to treat subclinical lymph node disease with chemotherapy using more effective regimens, the ability of PTV to cover high-risk vascular structures and lymph nodes in the periphery of the primary lesion, the predominance of LF and DF as the most common sites of recurrence, and the lower morbidity favor the adoption of IFRT.

Even patients undergoing surgery have high rates of LF (15%-50%) and DF (60%-90%) as the most common sites of failure. Hishinuma et al., in an analysis of autopsies of patients treated surgically, found LF in 75% of them, which was associated with perineural or lymphatic infiltration or extension to peripancreatic tissues. DF was also a common finding and was present in 75% of the patients, with a predominance of liver (50%) and peritoneum metastases (33%) [8]. Yu et al. conducted an extensive study mapping the recurrence pattern in 305 patients, 83 of whom presented with locoregional recurrence [9]. Most cases (77%) occurred near the superior mesenteric and celiac arteries. In patients treated with three-dimensional-conformal radiotherapy with IFRT, the incidental doses in these arteries and in peripancreatic and para-aortic lymph nodes were 80% and 40%-70% of the prescribed dose, respectively [10].

The occurrence of regional lymph node failure is a relatively rare event in the literature, especially when the treatment involves modern chemotherapy regimens based on gemcitabine or FOLFIRINOX. These are considered effective for controlling micrometastases in regional lymph nodes. Murphy et al. treated 74 patients with pancreatic tumors considered unresectable using a combination of gemcitabine (1000 mg/m^2^) and radiotherapy with a median dose of 36 Gy in 15 fractions using IFRT (GTV plus 1 cm of margins). With a median survival of 11.2 months, lymph node failure was observed in only four patients (5%), in three of whom the failure was located within the PTV and was similar to what was observed in the present study. Only one lymph node failure was located outside the PTV [11].

Treatments with IFRT result in a significant reduction in the volume of the stomach and small intestine compared with those with the elective inclusion of lymphatic drainage. A direct correlation was observed between PTV volume and gastrointestinal toxicity. Murphy et al. found a statistically significant association between high PTV and high morbidity. In the present study, the median PTV was 350 cm^3^ (range: 115-937 cm^3^). With the inclusion of elective lymph node drainage, the median PTV increased to 463 cm^3^ (range: 366-988 cm^3^), which would also lead to the inclusion of much higher volumes of organs at risk, especially the small intestine [11].

Based on this evidence, some authors and societies of the specialty have indicated specific volumes for locally advanced tumors, with the majority being favorable to IFRT treatments [12-14]. The American Society of Therapeutic Radiation Oncology recommends a total dose of 50.4-56 Gy, with concomitant chemotherapy for treatments performed using intensity-modulated radiotherapy (IMRT) or three-dimensional-conformal radiotherapy in conventional fractionations. For SBRT treatments, the volume recommendation is GTV with a small additional margin. With regard to the controversy about the volume to be used in conventional fractionations, the inclusion of elective drainage is conditionally recommended, along with a rigorous evaluation of the benefit in relation to the risks. In doing so, the uncertainty regarding the magnitude of the risks and the lack of consensus among the authors who elaborated the guidelines are recognized [12]. The European Society of Therapeutic Radiation Oncology guidelines do not recommend the elective inclusion of lymphatic drainage owing to the rarity of lymph node failure [13]. Huguet et al., in a French-American consensus, recommend a treatment regimen similar to the one used in the present study, with GTV encompassing the primary lesion and clinically positive lymph nodes, and an additional PTV of 1-2 cm in the anteroposterior and laterolateral axes and 2-3 cm in the craniocaudal axis [15]. These margins are indicated on the basis of studies that analyzed the mobility of the pancreas [16-20].

Dose

Local failure is a frequent outcome after treatment of locally advanced pancreatic tumor. Recent technical advances have been employed in an attempt to safely administer larger doses and improve local control rates.

SBRT offers the advantages of being performed with more precision and the biological effective dose being higher than that in conventional fractionations with fewer fractions. Planning done with more advanced techniques and treatments with a greater number of fractions have significantly reduced the complications and allowed treatment of larger volumes around the primary lesion [21-24].

It is still doubtful whether increasing the dose causes a corresponding increase in local control and overall survival. Studies using this technique have not been able to show significantly higher survival rates in relation to treatments performed using conventional fractionation [25-28]. Comparing SBRT and IMRT for unresectable pancreatic lesions was the objective of a study conducted at the Memorial Sloan-Kettering Cancer Center and the University of Colorado Cancer Center by Park et al. [28]. These authors analyzed 270 patients, of whom 44 were treated with SBRT and 226 with IMRT. SBRT was performed in five fractions, with a total dose of 30-33 Gy and IMRT in 25-28 fractions with a total dose of 45-56 Gy. With a median follow-up period of 12.9 months, the median overall survival was 15.7 months (95% CI: 12.8-17.8 months). Overall survival at one and two years was 56.2% and 25.7% for patients treated with SBRT, and 59.6% and 27.2% for those treated with IMRT, respectively. The cumulative LF incidence at one and two years was 34.4% and 48.7% for SBRT treatments and 30.2% and 45.5% for IMRT, respectively. For DF at one year, the values were 61.7% and 52.4% for these groups, respectively. None of these differences were statistically significant [28].

Moningi et al. analyzed the contemporary use of combined radiotherapy and chemotherapy treatments in 5,624 patients with locally advanced tumors. Owing to the availability of more therapeutic resources, especially with new systemic agents and better clinical selection of patients, these authors observed a decline in the use of radiotherapy from 55% to 45% among patients over 65 years of age during the period 2006-2013 and from 52% to 47% during the period 2006-2016 for younger patients. In both groups, there was an increase in the use of SBRT, more remarkable in recent years, of 10% and 12% for these groups of patients, respectively. Of the 2,522 patients over the age of 65 years, 53% underwent radiotherapy after induction chemotherapy, and 47% were treated with chemotherapy alone, with a predominance of gemcitabine-based regimens. Of the patients who received radiotherapy, 92% received conventional fractionations, with a median of 28 fractions. Similar results were observed in the group of younger patients, with 51% undergoing both chemotherapy and radiotherapy. The median survival was 11.5 months and 12 months, respectively, for patients undergoing radiotherapy with conventional fractionation and SBRT. These authors have identified greater selectivity in the use of radiotherapy in recent years. The results of this study also showed a reduction in the rate of complications owing to technological improvements observed in the treatments [29].

We carried out a retrospective analysis with a limited number of patients with a specific clinical situation. Probably, due to this, it was not possible to obtain a statistically significant correlation with the clinical and therapeutic variables in relation to the influence on local control or distant metastasis.

## Conclusions

The use of radiotherapy as a complementary therapy for patients with unresectable tumors after chemotherapy in carefully selected cases is a viable and safe option to improve local control rates and quality of life. In this study, IFRT was not correlated with significant lymph node therapeutic failure outside the limits of PTV and presented low morbidity rates. DF remained the predominant failure pattern.
